# Evaluation of Pharyngeal Airway Dimensions and Hyoid Bone Position in Children After Adenoidectomy or Adenotonsillectomy: A Cephalometric Study

**DOI:** 10.34172/joddd.2022.013

**Published:** 2022-10-15

**Authors:** Muhammed Hilmi Buyukavus, Ömer Faruk Sari, Gönül Kocakara

**Affiliations:** ^1^Department of Orthodontics, Faculty of Dentistry, Suleyman Demirel University, Isparta, Turkey; ^2^Dentist, Private Practice, AB Dental Health Center, Istanbul, Turkey

**Keywords:** Adenoidectomy, Cephalometry, Hyoid bone, Tonsillectomy

## Abstract

**Background.** The study aimed to compare the airway morphology and hyoid bone position in children undergoing adenoidectomy or adenotonsillectomy with healthy individuals with no skeletal problems in similar age and development period.

**Methods.** The subjects in the study were divided into three groups. These groups were defined as those having undergone adenoidectomy (53 children), those having undergone adenotonsillectomy (48 children), and the systemically healthy control group (63 children). Seventeen pharyngeal airway, nine hyoid, and four area measurements were used in the cephalometric radiographs of the children in all the groups. One-way analysis of variance was used to evaluate the children in each group. In addition, Tukey tests were used for a bilateral comparison of significant parameters. The results were considered statistically significant at *P*<0.05.

**Results.** The mean age of 164 patients in the study group was 12.76±2.85 years. The vertical airway length significantly decreased in the adenotonsillectomy group compared to other groups, while the epiglottic pharyngeal length significantly increased in the former than in the latter (*P*<0.05). The area measurements showed that although the oropharynx area significantly increased in the adenotonsillectomy group compared to other groups, the hypopharynx and total area measurements were significantly different compared with the control group (*P*<0.05). No statistically significant difference was found between the three groups in all measurements of the hyoid bone position (*P*>0.05).

**Conclusion.** The study showed that adenotonsillectomy operations caused more increases in the oropharynx and hypopharynx parts of the pharyngeal airway. Adenoidectomy and adenotonsillectomy operations did not contribute significantly to the hyoid bone position.

## Introduction

 The association between breathing problems (obstructive sleep apnea [OSA]) and abnormal dentofacial growth in children has been frequently reported in the literature. In growing humans, OSA usually occurs due to nasal and/or oropharyngeal obstruction from hypertrophic adenoids and tonsils, which is more prominent during childhood when the pharyngeal airway is not yet fully developed in terms of size.^1-3^For these reasons, adenotonsillectomy is considered a common procedure in children,^4,5^ with more than half a million procedures per year worldwide.^6^ In recent years, awareness about severe upper airway obstruction has increased due to its relationship with sleep-disordered breathing and especially OSA in children. There is evidence that children with severe disabilities need to be treated promptly and effectively. Although early identification of children with severe disabilities is recommended, the diagnosis of this type of respiratory dysfunction is sometimes delayed because parents are not aware of the problem.^7-9^

 The head and neck region is strategically important due to the close proximity of the stomatognathic system to the craniofacial region. The position of the hyoid bone provides important findings about the structural and positional relations of the head and neck region.^10^ However, the position of the hyoid bone is an important diagnostic tool in determining mouth breathing, detecting swallowing disorders, and evaluating facial types and dentofacial malformations.^11^ It is of great importance in the prognosis of orthodontic treatments and determining its recurrence after treatment. The position of the hyoid bone also changes with age. King followed the position and growth of the hyoid bone from the 3rd month to 16 years of age in 1952, observing that the hyoid bone is located at a level between the 3rd and 4th cervical vertebrae in children and above the symphysis, and in adults at the level of the 4th cervical vertebra and below the symphysis. It has been shown that the downward movement of the hyoid bone is quite rapid in infancy and early childhood, and then this movement slows down.^12^

 Limited data are available on patients with hypertrophic tonsils and adenoids, pharyngeal airway dimensions, hyoid bone position, changes in craniofacial morphology, and changes in pharyngeal airway dimensions before and after adenoidectomy, tonsillectomy, and adenotonsillectomy operations. However, no studies have compared the effects of surgeries on pharyngeal airway and hyoid bone position using a control group.

 Based on the information in the literature that the airway morphology and hyoid bone position might change after adenoidectomy and adenotonsillectomy procedures, this study aimed to compare the airway morphology and hyoid bone position of children undergoing adenoidectomy or adenotonsillectomy with that of healthy individuals without skeletal problems in the similar age and growth and development stage.

## Methods

 Permission to start this clinical study was obtained from the ethics committee of the university, and informed consent was obtained from the parents of the patients included in the study before the study. Lateral cephalometric radiographs of patients referring to our clinic for orthodontic treatment due to skeletal or dental malocclusions were obtained at the beginning of the treatment. The patients were divided into three groups. These groups were defined as those who had undergone adenoidectomy, those who had undergone adenotonsillectomy, and the systemically healthy control group. The minimum sample size required for the study was calculated at n = 21 in each group based on an alpha error probability of 0.05 and the upper pharyngeal airway length at 80% power.^13^ More individuals were included to increase the power of the study (90%).

 The anamnesis forms were checked for any craniofacial anomalies or systemic disorders and airway pathologies in the individuals included in the study. In addition, individuals with cephalometric radiographs with inadequate imaging quality (radiography with artifacts, radiographs with incorrect head position, etc.) and individuals who had previously received orthodontic treatment were excluded from the study. A total of 605 patients were evaluated for the study, and 164 subjects, 80 girls and 84 boys, were included.

 Individuals meeting the inclusion criteria were divided into groups at the beginning of the treatment according to the anamnesis forms, including the medical history taken during the first visit to our clinic. Finally, of 101 individuals having undergone adenoidectomy and/or tonsillectomy procedures, 53 who had undergone only adenoidectomy were assigned to group 1, and 48 who had undergone adenotonsillectomy were assigned to group 2. Sixty-three children with skeletal Cl I malocclusion and normal growth pattern and no respiratory problems, who had not undergone tonsillectomy or adenoidectomy procedures and were considered healthy, were assigned to group 3 (control group). Those in the third group were examined by comparing pharyngeal airway and hyoid measurements.

###  Cephalometric analysis

**Figure 1:**
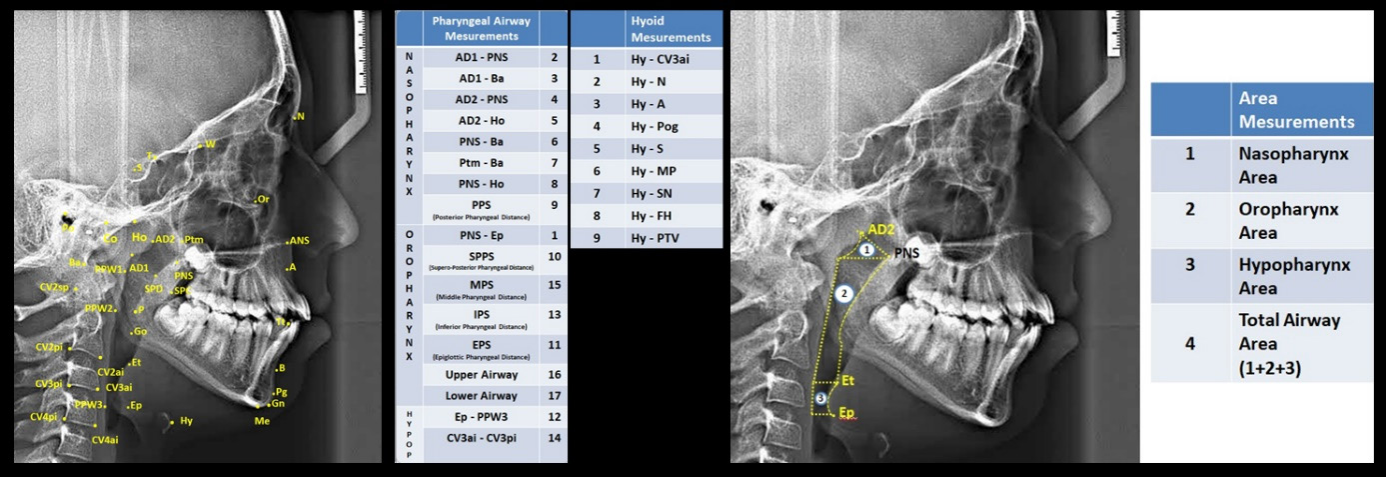
Cephalometric landmarks and all measurements used in this study (PPS: palatal pharyngeal distance: on the line passing from PNS, parallel to FH. SPSS, superior posterior pharyngeal distance: on the line passing from the midpoint of the soft palate, parallel to FH. MPS, middle pharyngeal distance: on the line passing from P, parallel to FH. IPS, inferior pharyngeal distance: on the line passing from cv2a, parallel to FH. EPS, epiglottic pharyngeal distance: on the line passing from E, parallel to FH).

 NemoCeph cephalometric software program (NX 2009 for Windows, Nemotec, Madrid, Spain) for linear measurements on cephalometric radiographs and SketchAndCalc software program for area measurements (SketchAndCalc Area Calculation software, Axiom Welldone, https://www.sketchandcalc.com/) were used. In this study, 35 cephalometric points, 8 cephalometric planes, and 30 cephalometric measurements (5 craniofacial, 8 nasopharyngeal, 7 oropharyngeal, 2 hypopharyngeal, 9 hyoid measurements, and 4 area measurements) were used (Figure 1).

###  Statistical analysis

 Kolmogorov-Smirnov test was used to determine the distribution of data. Parametric tests were used because the majority of the measurements used in the study were homogeneously distributed. The distribution of sex and growth/development stages of the individuals in the study were compared with Pearson’s chi-square test. One-way ANOVA was used to compare the airway and hyoid measurements of the groups. Post hoc Tukey tests were used for two-by-two group comparisons of the groups. For the reliability of the measurements, 50 films randomly selected from 164 cephalograms were repeated by the researchers. Cronbach’s alpha repeatability coefficients determined for each measurement were found to be high (α ≥0.863). SPSS (SPSS for Windows, version 21.0; SPSS Inc., IL, USA) was used for data analysis.

## Results

Table 1 presents the distribution of the groups regarding age, sex, and growth and development stage. No statistically significant relationship was found between groups in chronological age, sex, and growth and development stages (*P* > 0.05), indicating that the groups had similar characteristics and were well matched demographically.

**Table 1:** Demographic statistics by groups

		**Group 1**	**Group 2**	**Group 3**	**Total** **(n=164)**	***P*** ** value**
		**Adenoidectomy** **(n=53)**	**Adenotonsillectomy** **(n=48)**	**Control** **(n=63)**		
Chronological Age (Mean ± SD)		12.61 ± 2.66	12.71 ± 2.38	12.56 ± 2.61	12.76 ± 2.85	0.123^a^
Sex, No. (%)	Boys	28 (52.83)	23 (47.92)	33 (52.38)	84 (100)	0.237^b^
	Girls	25 (47.17)	25 (52.08)	30 (47.62)	80 (100)	
Growth development period, No. (%)	Pre-peak	16 (30.18)	18 (37.5)	22 (34.92)	56 (100)	0.330^b^
	Peak	23 (43.39)	18 (37.5)	28 (44.44)	69 (100)	
	Post-peak	14 (26.43)	12 (25)	13 (20.64)	39 (100)	

^a^ One-way analysis of variance (ANOVA); ^b^ Pearson chi-square test.

 The nasopharyngeal airway measurements showed no significant differences between the groups (*P* > 0.05; Table 2). However, significant differences were found in the oropharyngeal measurements of the groups. Although vertical airway length (PNS-Ep) measurement significantly decreased in the adenotonsillectomy group than in the other two groups, the epiglottic pharyngeal length and McNamara’s lower airway length increased significantly in the other two groups (*P* < 0.05; Table 2). The hypopharyngeal measurements revealed a significant difference in CV3ai-CV3pi measurements between the adenotonsillectomy and control groups (*P* < 0.05).

**Table 2:** Comparison of pharyngeal airway and hyoid measurements according to groups

			**Group 1** **Adenoidectomy**	**Group 2** **Adenotonsillectomy**	**Group 3** **Control**	**Post hoc** **tests**			**ANOVA**
			**Mean±SD**	**Mean±SD**	**Mean±SD**	**1-2**	**1-3**	**2-3**	***P*** ** value**
Pharyngeal airway measurements	Nasopharynx	AD1-PNS	23.02 ± 5.61	22.03 ± 4.98	23.04 ± 6.00	NS	NS	NS	0.561
		AD1-Ba	19.77 ± 4.17	19.83 ± 4.34	18.90 ± 3.64	NS	NS	NS	0.668
		AD2-PNS	18.09 ± 4.67	17.51 ± 4.67	18.42 ± 4.19	NS	NS	NS	0.671
		AD2-Ho	11.31 ± 4.10	11.31 ± 4.36	10.93 ± 3.49	NS	NS	NS	0.929
		PNS-Ba	42.49 ± 5.01	42.47 ± 5.48	42.00 ± 5.83	NS	NS	NS	0.934
		Ptm-Ba	37.96 ± 3.61	37.98 ± 3.89	36.77 ± 4.63	NS	NS	NS	0.448
		PNS-Ho	29.41 ± 4.21	29.09 ± 2.67	28.74 ± 4.55	NS	NS	NS	0.762
		PPS	24.40 ± 6.26	23.28 ± 4.92	23.78 ± 6.19	NS	NS	NS	0.568
	Oropharynx	PNS-Ep	55.92 ± 7.17	45.10 ± 3.40	55.06 ± 6.93	***	NS	***	**0.000**
		SPPS	9.88 ± 3.02	9.87 ± 2.66	10.33 ± 2.50	NS	NS	NS	0.773
		MPS	11.38 ± 3.93	12.29 ± 3.23	10.34 ± 2.67	NS	NS	NS	0.073
		IPS	12.19 ± 4.96	13.38 ± 5.80	11.05 ± 4.91	NS	NS	NS	0.194
		EPS	9.86 ± 3.07	10.89 ± 2.65	9.43 ± 2.22	NS	NS	*	**0.049**
		Upper airway	6.58 ± 2.60	6.71 ± 2.26	6.79 ± 2.50	NS	NS	NS	0.936
		Lower airway	8.99 ± 2.57	11.42 ± 6.75	8.37 ± 2.11	*	NS	*	**0.010**
	Hypopharynx	Eb-PPW3	12.76 ± 4.15	13.95 ± 3.06	12.37 ± 4.45	NS	NS	NS	0.124
		CV3ai-CV3pi	9.09 ± 3.80	10.27 ± 2.82	8.31 ± 2.54	NS	NS	*	**0.029**
Hyoid measurements		Hy – CV3ai	31.62 ± 10.25	30.66 ± 4.40	28.79 ± 8.00	NS	NS	NS	0.374
		Hy - N	112.34 ± 16.59	111.40 ± 12.66	106.11 ± 13.2	NS	NS	NS	0.328
		Hy - A	69.99 ± 6.92	68.31 ± 7.19	68.29 ± 7.09	NS	NS	NS	0.401
		Hy - Pg	43.10 ± 6.54	44.95 ± 5.80	41.86 ± 6.39	NS	NS	NS	0.094
		Hy - S	94.09 ± 10.60	91.20 ± 10.37	93.16 ± 9.10	NS	NS	NS	0.314
		Hy - MP	13.71 ± 15.44	12.16 ± 5.95	11.43 ± 3.56	NS	NS	NS	0.627
		Hy - SN	94.85 ± 10.10	92.62 ± 9.37	93.70 ± 9.12	NS	NS	NS	0.463
		Hy - FH	75.90 ± 8.13	71.92 ± 14.37	73.89 ± 7.64	NS	NS	NS	0.178
		Hy - PTV	-1.26 ± 6.58	-0.16 ± 6.07	-0.89 ± 5.51	NS	NS	NS	0.630
Area measurements		Oropharynx area	408.16 ± 123.71	449.86 ± 129.19	399.1 ± 109.7	**	NS	**	**0.000**
		Nasopharynx area	121.35 ± 66.22	118.88 ± 44.23	122.2 ± 67.19	NS	NS	NS	0.961
		Hypopharynx area	199.81 ± 65.98	199.81 ± 86.54	177.97 ± 58.2	NS	*	*	**0.006**
		Total area	729.33 ± 197.17	738.99 ± 211.05	709.35 ± 185	NS	**	**	**0.000**

SD: Standard Deviation; NS: Not-significant *P* > 0.05 *P < 0.05 **P < 0.01

 The area measurements showed that the oropharynx area significantly increased in the adenotonsillectomy group compared with the other two groups; hypopharynx and total area measurements were significantly different from the control group (*P* < 0.05; Table 2). No significant difference was found between the three groups in all measurements of the hyoid bone position (*P* > 0.05; Table 2).

## Discussion

 Adenoidectomy, tonsillectomy, and adenotonsillectomy are the most common surgeries performed in children because they have many indications, and the symptoms affect the growth and development of the child directly or indirectly. Therefore, tonsillectomy and adenoidectomy procedures are believed to affect growth and development positively.^14,15^OSA prevents growth and development in children. Growth and development have been reported after adenotonsillectomy due to OSA.^16-18^ For this reason, the effects of craniofacial growth on dentoalveolar and craniofacial structures have been investigated by orthodontists in a limited number of studies.

 Becking et al^19^ examined the effects of adenotonsillectomy on dentofacial development. Numerous studies have shown that hypertrophic adenoids and tonsils may be risk factors for dentofacial deformations, and adenotonsillectomy normalizes dentofacial development. This reflects the assertion that nasopharyngeal airway obstruction may be a factor involved in the development of dentofacial deformities. In addition, studies have shown normalization and transverse improvement in the inclination of the upper and lower incisors after adenotonsillectomy and normalization in the horizontal growth pattern of the mandible. Airway morphology and hyoid bone position have been examined many times by orthodontists because it is closely related to craniofacial morphology. Many skeletal and dentoalveolar disorders are encountered in children with airway problems. The morphology is also positively affected after the respiratory problems in children are corrected in the early stages.

 Petraccone Caixeta et al^20^ showed a significantly different pattern of dental arch development in children undergoing adenotonsillectomy compared with untreated controls. They reported increased maxillary transverse development after adenotonsillectomy. Krasny et al^21^ demonstrated that the flow of the nasopharyngeal airway below 38% should be an indication for adenoidectomy; otherwise, the condition is associated with increased severity of malocclusion in children with orthodontic problems. In addition, a significant increase in the physiological development, psychosocial development, and quality of life of children has been reported after adenotonsillectomy.^22-24^

 The cephalogram, a sagittal x-ray of the head and neck, is the most widely used examination, especially in orthodontics. It is a simple, affordable, easily accessible, and repeatable way to diagnose airway problems. A high correlation was found between posterior rhinoscopy results and the dimensions of adenoids in the posterior nasopharyngeal wall seen on lateral radiographs.^2,25^ LCR has been reported by many studies to be a convenient method to measure adenoid hypertrophy in children aged 6–12 years, owing to its ease of use and low cost.^26^ Therefore, it was used for airway measurements in our study.

 No significant changes were observed in nasopharyngeal dimensions in this study. Although the nasopharyngeal part of the airway increased in children after adenoidectomy and adenotonsillectomy procedures, there was no statistical difference. Although airway changes were examined before and after adenoidectomy and adenotonsillectomy procedures in earlier studies, a direct comparison could not be made with the findings of this study because no comparative studies were encountered. The current study would be a guide for future studies.

 De Magalhães Bertoz et al^27^ reported that airway volume increased in 30 individuals with an average age of 8.9 years who underwent adenotonsillectomy; however, no correlation was observed between the magnitude of anatomical changes and an improvement in respiration. The oropharynx and hypopharynx improved significantly after adenotonsillectomy compared with those in both healthy children and those who underwent only adenoidectomy in this study. The tonsils were larger in volume than adenoids, and the resection of the hypertrophic tonsils significantly increased the airway in the oropharynx. Area measurements also supported our linear findings.

 Cone-beam computed tomography provided a three-dimensional evaluation of the airway by volume. However, this was not preferred because of radiation dose, cost, and unsuitability for routine use. This situation could be considered as the limitation of our study. The findings of this study were supported by two-dimensional digital area measurements that were of little use in the literature to minimize this disadvantage. Adenoidectomy, tonsillectomy, and adenotonsillectomy procedures, the age at which the procedure was performed, the number of years before the surgery, and preoperative findings were ignored because this study was designed as a retrospective study, and information about patient anamnesis forms was lacking. This situation could be considered another limitation of this study.

## Conclusions

 This study showed that adenoidectomy and adenotonsillectomy procedures contributed positively to the pharyngeal airway dimensions. Adenotonsillectomy caused greater increases in the oropharynx and hypopharynx parts of the pharyngeal airway. Adenoidectomy and adenotonsillectomy did not significantly change the hyoid bone position.

## Author’s Contribution

 MHB: Supervision, conceptualization, data curation, investigation, writing the original draft, review, and editing. GK: Conceptualization, data curation, formal analysis, investigation, methodology, writing the original draft, review, and editing. ÖFS: Formal analysis, software, writing the original draft.

## Funding

 Self-funded.

## Ethics Approval

 This study was approved by the Clinical Research Ethics Committee, Suleyman Demirel University. Ethical approval number: 16.01.2019-7.

## Competing Interests

 None.
