# Correction to: Evaluation of pharyngeal airway dimensions and hyoid bone position in children after adenoidectomy or adenotonsillectomy: A cephalometric study

**DOI:** 10.34172/joddd.2022.044

**Published:** 2022-12-30

**Authors:** Muhammed Hilmi Buyukcavus, Ömer Faruk Sari, Gönül Kocakara

**Affiliations:** ^1^Department of Orthodontics, Faculty of Dentistry, Suleyman Demirel University, Isparta, Turkey; ^2^Dentist, Private Practice, AB Dental Health Center, Istanbul, Turkey

In the article entitled "Evaluation of Pharyngeal Airway Dimensions and Hyoid Bone Position in Children After Adenoidectomy or Adenotonsillectomy: A Cephalometric Study" which appeared in J Dent Res Dent Clin Dent Prospect 2022;16(2): 81-86. doi: 10.34172/joddd.2022.013, the name of the first author was misspelled. The correct name of the first author is Muhammed Hilmi Buyukcavus. The original version of the article has been updated to reflect these corrections.

